# Hospital Schools During COVID-19: Teachers' Perspective

**DOI:** 10.34763/jmotherandchild.20212503SI.d-21-00016

**Published:** 2022-02-09

**Authors:** Maja Gajda, Aleksandra Berkowska, Agnieszka Małkowska-Szkutnik

**Affiliations:** 1 Faculty of Education, Warsaw University, Warsaw, Poland

**Keywords:** Remote teaching, hybrid teaching, hospital teachers, COVID-19 pandemic, in-hospital education

## Abstract

Temporary lockdowns have been introduced in many countries as a preventive measure against the spread of the virus in 2020 and 2021. School closures and remote education have posed some difficulties for both students and teachers. A qualitative study and the semi-structured interview method was chosen to collect hospital teachers’ insights into their work experiences during the pandemic. The sample consisted of 21 participants who worked as hospital school teachers. The study revealed the following thematic areas: introduction of remote/hybrid teaching (Frequency=8), lack of the sense of employment stability (F=4), limited contact with students (F=6), necessity to adapt to dynamically changing conditions (F=3), sedentary character of work (F=3), improvement of the quality of work and work conditions (F=4). The research was conducted as part of the Back to School Project (project number: 2019-1-PL01-KA201-065602), with funding from Erasmus+. The data collected during the study will be used to create guidebooks for both hospital school and mainstream school teachers.

## Background

The COVID-19 pandemic has had an impact on people’s lives across the world. Temporary lockdowns have been introduced in many countries as a preventive measure against the spread of the virus in 2020 and 2021. Many governments have decided to close schools, and remote education replaced traditional lessons at school. It is estimated that more than 1.5 billion students from schools or universities around the world have been affected by the disruption of formal education [1].

School closures and remote education have posed some difficulties for both students and teachers [2, 3, 4]. Remote education has resulted in increased screen time [5, 6, 7, 8], and many students and teachers have become vulnerable to the overuse of digital devices. Symptoms of such overuse include irritability, fatigue, back pain, and eye problems [9, 10, 11, 12]. Another risk is connected with insufficient physical activity that can be partially responsible for developing obesity in children and adolescents [13]. Moreover, teachers and students’ mental health was also at risk because replacing direct human interactions with online contact may trigger feelings of loneliness and isolation [15]. In addition to the above-mentioned risks, during the COVID-19 pandemic teachers have also reported decreased job satisfaction [15, 16], being overloaded with work [15, 17], and experiencing more work-related stress than before the pandemic [18]. In general, teachers’ quality of life seems to have worsened during the COVID-19 pandemic [19, 20].

Polish hospital schools located in hospitals and healthcare resorts have been closed several times during the pandemic. In the beginning, there was only remote education and teachers could not enter hospitals due to strict preventive measures. After the initial closure and distance learning, hybrid teaching was also introduced in accordance with the regulations of the Minister of Education. For many Polish teachers, it seemed to have been the first time they used digital education tools in their work on such a large scale [15].

Researching the area related to hospital education is valid because hospital teachers play an important role in children and adolescents’ school success [21], school engagement [22, 23], and general wellbeing [24]. They also support students who often experience learning difficulties caused by illness or hospitalization [25, 26] and ensure the continuity of their education. Moreover, hospital teachers play a role in keeping students active as they provide them the opportunity to engage and socialize with peers [26]. A hospital school is a source of normality in children and adolescents’ hospital life and a stepping stone from hospitalization [22, 24, 27, 28]. However, at the moment of the publication of this article, a very limited number of studies focusing on hospital teachers’ work during the pandemic was available. Perhaps it is because of the difficult and dynamic situation; relatively little time has passed after the first COVID-19 outbreaks. Also, researchers show lower interest in hospital teachers as a professional group because they constitute only a small percentage of all teachers [29, 30].

The aim of this study was to learn more about the hospital teachers’ perceptions of their work during the pandemic, problems they were facing, and the needs they had in that difficult period.

## Material and methods

The research was conducted as part of the Back to School Project (project number: 2019-1-PL01-KA201-065602), with funding from Erasmus+. It acquired a positive opinion from the Bioethical Committee of the Institute of Mother and Child (number 23/2020, 21.05.2020). The data collected during the study will be used to create guidebooks for teachers from hospital schools and regular schools. The outputs will be available online on the website of BTS Project (https://backtoschoolproject.eu/) in autumn 2021. Also, various innovative materials will be created for teachers, parents, and students with special educational needs, such as chronic conditions.

A qualitative study was chosen to collect hospital teachers’ insights into their work experience during the COVID-19 pandemic. Findings presented in this article include only part of the interview: that related to the COVID-19 pandemic. There were also questions about hospital teachers’ work from pre-pandemic times. However, the results from this part of the study will be published in a separate publication.

The semi-structured interview method was chosen because it allows the researchers to develop “in-depth accounts of experiences and perceptions with individuals” [p. 71 in item 31 References], ask them follow-up questions, and explore unexpected threads. It is worth mentioning that this method may not ensure objectivity, as it is used for gathering personal perspectives instead [31].

## Participants’ characteristics

There were 21 teachers who took part in the interviews. They were employed in schools located in medical facilities. Participants included 18 in-hospital school teachers and 3 healthcare resort school teachers. All 21 participants met the study inclusion criteria:

- being employed in a hospital school or healthcare resort school at the time of the interview;
- having at least one year of work experience in a hospital/healthcare resort setting;
- having at least a contractual teacher’s degree of professional advancement concluding with an official examination.

The age of the participants ranged from 28 to 58 years. There were 19 female teachers and 2 male teachers. The participants had varied experiences and worked with students from different educational stages. Among the participants were preschool, primary, and secondary school teachers, as well as educators.

## Organisation of the study, data collection and methods of analysis

Because of the pandemic-related restrictions introduced in the whole country, the researchers were able to contact only 48 hospital schools. From 21 teachers who volunteered, all met the criteria of inclusion and took part in the interviews. Informed consent of personal data processing was obtained from the participants.

Due to the COVID-19 pandemic, the interviews were conducted online. Three group interviews were conducted: one on 8 June 2020, and the following two on 17 November 2020. Each interview lasted approximately an hour and a half. During the first interview, hospital schools and healthcare resort schools were closed and teachers worked remotely. However, during the next two interviews, some teachers were on a hybrid schedule.

The interviews were recorded and then transcribed. The collected data was analysed with a two-step coding procedure [32]. In the analysis, the main areas corresponding with the research questions emerged, and descriptive codes were assigned to them by two independent researchers. ATLAS.ti. 9, a program for qualitative analysis, was used for that purpose. No outcomes were assumed a priori [32].

## Results

During the first stage of thematic analysis, six areas emerged. Table 1 shows the frequency of given codes within each area.

**Table 1:** Hospital teachers’ perceptions of changes in work conditions resulting from the COVID-19 pandemic.

Theme	Frequency
Introduction of remote/hybrid teaching	8
Lack of the sense of employment stability	4
Limited contact with students	6
The necessity to adapt to dynamically changing conditions	3
Sedentary character of work	3
Improvement of the quality of work and work conditions	4
Total	28

The quotations below are marked with abbreviations from T1 (Teacher 1) to T21, which were assigned to interview participants.

## Introduction of remote/hybrid teaching

The first area that was analysed refers to the change in the work organisation (F=8). During the COVID-19 pandemic, hospital schools have worked online, which means that teachers stayed at home and taught synchronous or asynchronous lessons. They have also taught on a hybrid schedule, which means that some lessons took place in hospitals and the rest of them were held online. These changes in work organisation were introduced due to national regulations restricting personal contact of students and teachers. One of the participants described their experience in the following way:

*Some time ago we had a hybrid classroom, some of us worked in hospitals, some of us worked from home. Then, each of us could work only in one ward and we couldn’t go from one ward to another. Now, they told us not to come at all because there have been a lot of new cases* (T7).

## Lack of the sense of employment stability

The interviewees pointed out that the COVID-19 pandemic has had an impact on their sense of job stability and the safety of employment. The pandemic has intensified such concerns (F= 4), especially in healthcare resort school teachers. One of the participants raised a problem with local governments' plans to transform their school into a center for the elderly. Other participants also said that there had been plans to close their schools for a long time, and during the pandemic, those plans became more realistic than before. This area is reflected in the following statements:

*We've heard that they want to close our facility for a long time now, but recently, due to the COVID-19 pandemic, fewer children are coming, and they started talking about turning our sanatorium into a retirement home, and that hurts us a lot. We don't know what will happen next* (T5).

*We are afraid of losing our jobs. If our school was closed, we would not find work anywhere else, because we are the only healthcare resort school that exists in our region* (T12).

## Limited contact with students

Another difficult part for the teachers was limited contact with students or complete lack of that contact (F=6). This area corresponds with the previously mentioned changes in work organisation resulting in school closures and contact restrictions. Most participants perceived this situation in a negative light:

*I can say something from the perspective of a preschool teacher who fell out of the system. The work of educators and preschool teachers has moved completely outside the hospital. Contact with students is very limited* (T11).

Moreover, several participants pointed out that the shift to remote or hybrid teaching harmed younger students in particular. They had concerns that the lack of personal contact would worsen students’ learning opportunities and hinder establishing student-teacher relationships. This area was described by participants as follows:

*There is no substitute for a personal relationship, especially with younger learners. Now, I have lessons only with first graders. We're learning letters and numbers. Personal contact is very important for that* (T7).

## The necessity to adapt to dynamically changing conditions

Several teachers pointed out that the COVID-19 pandemic forced them to adapt to dynamically changing work conditions (F=3). For some of them, it was their first contact with digital educational tools. Also, when hospital teachers could not meet with students, they devoted themselves to creating educational aids for future in-class lessons. This way of working was reported mostly by preschool teachers who were unable to meet students for a long time during the pandemic.

*We switched to a different style of work. Now, we're working more on educational materials. We're developing our skills and getting ready for the next hospital entry. This element is very difficult, I must say* (T11).

*Now, our job is mainly about creating educational sheets and handouts that the kids can use later* (T14).

*Our work is limited to the preparation of materials for patients. Worksheets, handouts, various materials based on brainstorming, multimedia presentations, etc*. (T15).

## Sedentary character of work

Another difficulty faced by teachers was the necessity to spend many hours in front of the computer (F = 3). Thus, some of the teachers reported the need to return to traditional lessons.

*I would like this to change. Now I spend my days in front of the computer screen* (T11).

*All days in front of the computer – it is difficult for me* (T6).

*We keep sitting all day in front of the laptop* (T9).

## Improvement of the quality of work conditions

In contradiction to the above-mentioned difficulties connected with work conditions, teachers also pointed out that there were some positive sides to the situation. As a result of the changes in the work organisation, hospital teachers could spend more time with individual students during a remote lesson, compared to working in the hospital ward (F = 4). Several teachers said that they appreciated the possibility to start lessons in hospitals, and then continue them remotely. This area is reflected in the following statements:

*I was constantly getting frustrated that I didn't get to someone. Now, I have activities scheduled with children. Well, at least with those I can contact. And now I have lessons with them three times a week, often one hour per child. So, I just love the fact that I can plan something and actually carry out this lesson, and that I can spend so much time with these children and devote real time to them and not struggle constantly just to reach everyone* (T6).

*Now we have direct contact with children and if we don’t manage to do something in the hospital, we can finish it later online at home. In the afternoon, when we connect with children, there are no more treatments, medical examinations, and it is easier to work then* (T10).

*I really appreciate both online contact and being with students in a hospital. And hybrid teaching gives us such a great opportunity. It's wonderful because I can have contact with students, and then, I can continue our work online*. (T9).

## Discussion

The article presents the results from the study conducted with 21 Polish hospital school teachers in 2020, during the COVID-19 pandemic. The interviewed teachers pointed to the changes in work organisation caused by the pandemic, which had an impact on the quality of their work.

The social distancing restrictions introduced because of COVID-19 have intensified sedentary and screen-related behaviours [5, 6, 7, 8]. Because of the shift in work conditions, hospital teachers started working long hours in front of the computer, which exposed them to the risk of irritability and health problems such as eye strain, back pain, and so on. In the study, several participants pointed out that they had been experiencing similar symptoms, and most of them described sitting in front of the computer as difficult and expressed a wish to return to traditional face-to-face teaching methods.

Another important area that emerged in the study is connected with hospital teachers’ professional training and their experience in e-learning methods. In general, when the pandemic struck, many teachers had to learn how to use online tools. They were not prepared for remote teaching and were left without much methodological support [15]. Some of the interviewed hospital teachers revealed that they had not worked remotely with students before the pandemic. However, it is worth mentioning that in many countries remote education had been widely used in hospital schools long before the pandemic outbreak [28, 33, 34]. It seems that remote teaching solutions, introduced on a large scale in Poland because of the pandemic, could be considered by Polish officials and hospital teachers as an alternative to traditional lessons in hospital schools. However, it is recommended that online tools should be used as a supplement only and not as a substitute, because remote learning requires high self-motivation from students [30].

In general, hospital teachers perceive their work as demanding organisational flexibility [27, 37]. They need to constantly adjust their lesson plans to changing conditions of students’ health and interruptions caused by medical procedures. Moreover, they work with students of varied age and educational advancement and teach highly individualised lessons at the bedside or in recreational rooms. All in all, hospital teachers perceive their work conditions in a hospital setting as difficult. An interesting finding from the study is that for some interviewed hospital teachers, the introduction of remote teaching had improved work conditions and quality of lessons. They suggested that thanks to remote tools they were able to devote more time to individual students and plan lessons in advance. Also, they perceived hybrid teaching as the most convenient solution because it enabled them to have face-to-face contact with students and continue lessons with them in an online form.

Teachers from hospital schools perceive personal contact with students as very important, especially considering that they usually teach one-on-one lessons at the bedside . When hospital schools in Poland were closed due to national regulations, teachers’ contact with students was very limited. The interviewed teachers perceived this situation as difficult and frustrating. They also expressed their concern mainly about younger learners and their abilities to deal with the situation. Hospital teachers’ overall attitude, characterised by feeling responsible for supporting students' well-being and educational goals [37], may have intensified the frustration caused by the limitations in teacher-student contact.

The sudden situation related to the outbreak of the pandemic put teachers at risk of high stress [18,35,36]. On one hand, working on a hybrid schedule and entering hospital wards during the pandemic could have resulted in an additional stress load for hospital teachers. On the other hand, the lack of contact with students also was a source of mental strain and frustration for teachers. They perceive personal contact as especially important because they usually teach one-on-one lessons at the bedside [27, 37, 38, 39]. They also expressed their concerns about younger learners and their abilities to deal with the situation. Moreover, hospital teachers had to adapt to dynamically changing conditions and develop skills necessary for remote education. All in all, they have experienced many changes in a short period of time and had little support, which was a stressful experience. It is worth mentioning that even before the pandemic, hospital teachers had perceived their work as stressful because of emotionally difficult situations connected with a student's death, sudden deterioration of health, and working under the pressure of constant flexibility and unpredictable conditions [27, 37]. More studies are needed to analyse the changes in needs and support for hospital teachers during and shortly after the COVID-19 pandemic.

One of the strengths of the study is that it was carried out during the COVID-19 pandemic, which enabled researchers to gather relevant and current information on hospital teachers’ perceptions of the situation. Thus, the results of the research are up-to-date and provide relevant insight into hospital teachers’ reflections, attitudes, needs, and perceived difficulties resulting from this unprecedented situation.

The study has several limitations. The main limitation was the pandemic situation which had a negative impact on the recruitment of participants to the study. Moreover, the researchers could not control the environment in which participants were at the moment of conducting the interviews. Also, the study was limited to Polish hospital teachers only so the results and conclusions should not be generalised to other populations. Due to the qualitative nature of the study, the results should not be treated as the only possible perspective on hospital teachers’ situation during the pandemic.

## 5 Conclusion

Hospital teachers have an important role in ensuring the continuity of education in students with chronic or long-lasting conditions [22,27]. Despite the importance of their job, it seems to have been marginalised and underrated [30,40,41]. Gathering insights into the perceptions of hospital teachers’ work during such challenging and unpredictable times as the pandemic adds a valuable perspective to the existing studies.

The COVID-19 pandemic has posed a challenge for many teachers [42]. For hospital teachers the difficulties connected with school closures and lack of contact with students were also a source of stress [36]. However, hospital teachers had varied perspectives on the introduction of distance and hybrid teaching during the pandemic. The majority of educators and preschool teachers perceived these solutions in a negative light. They highlighted that remote teaching made it practically impossible to organise activities they usually did with students because they required face-to-face interaction. However, some teachers also pointed to some positive aspects of the situation. For example, subject teachers reported that they were able to spend more time with individual students, which had a positive impact on the quality of work and job satisfaction. Moreover, although remote education has been widely available as one of the teaching methods in hospital schools in many countries [30,33,34], at least for some Polish hospital teachers it was a novelty. However, it seems that this way of teaching has its potential in a hospital environment, especially in older students’ education as a supplementary method.

There is a further need to analyse the current conditions and various aspects of hospital teachers' work, to see the advantages and disadvantages of the new situation, and to take measures to minimise the negative impact of the pandemic on the well-being of teachers.

## References

[1] (2021). Supporting Learning Recovery One Year Into COVID-19: The Global Education Coalition in Action. UNESCO; Global Education Coalition.

[2] Cachón-Zagalaz J, Sánchez-Zafra M, Sanabrias-Moreno D, González-Valero G, Lara-Sánchez AJ, Zagalaz-Sánchez ML (2020). Systematic Review of the Literature About the Effects of the COVID-19 Pandemic on the Lives of School Children. Front Psychol.

[3] Pokhrel S, Chhetri R (2021). A Literature Review on Impact of COVID-19 Pandemic on Teaching and Learning. High Educ Futur.

[4] Korzycka M, Bójko M, Radiukiewicz K, Dzielska A, Nałęcz H, Kleszczewska D, Małkowska-Szkutnik A, Fijałkowska A (2021). Demographic analysis of difficulties related to remote education in Poland from the perspective of adolescents during the COVID-19 pandemic. Ann Agric Environ Med.

[5] (2020). Excessive Screen Use and Gaming Considerations During COVID19.

[6] Schmidt SCE, Anedda B, Burchartz A, Eichsteller A, Kolb S, Nigg C (2020). Physical activity and screen time of children and adolescents before and during the COVID-19 lockdown in Germany: a natural experiment. Sci Rep.

[7] Okely AD, Kariippanon KE, Guan H, Taylor EK, Suesse T, Cross PL (2021). Global effect of COVID-19 pandemic on physical activity, sedentary behaviour and sleep among 3- to 5-year-old children: a longitudinal study of 14 countries. BMC Public Health.

[8] Kovacs VA, Starc G, Brandes M, Kaj M, Blagus R, Leskošek B (2021). Physical activity, screen time and the COVID-19 school closures in Europe - An observational study in 10 countries. Eur J Sport Sci.

[9] (2014). Public health implications of excessive use of the internet, computers, smartphones and similar electronic devices: meeting report, Main Meeting Hall, Foundation for Promotion of Cancer Research.

[10] Sarla GS (2019). Excessive Use of Electronic Gadgets: Health Effects. Egypt J Intern Med.

[11] (2020). Screen Time 2020 Report: attitudes and opinions of employers and eye care providers about screen time and blue light.

[12] Wong CW, Tsai A, Jonas JB, Ohno-Matsui K, Chen J, Ang M (2021). Digital Screen Time During the COVID-19 Pandemic: Risk for a Further Myopia Boom?. Am J Ophthalmol.

[13] Dunton GF, Do B, Wang SD (2020). Early Effects of the COVID-19 Pandemic on Physical Activity and Sedentary Behavior in Children Living in the U.S. BMC Public Health.

[14] Hu T, Zheng X, Huang M (2020). Absence and Presence of Human Interaction: The Relationship Between Loneliness and Empathy. Front Psychol.

[15] Ptaszek G, Stunża GD, Pyżalski J, Dębski M, Bigaj M (2020). Edukacja zdalna: co stało się z uczniami, ich rodzicami i nauczycielami? (Remote education: what happened to students, their parents and teachers).

[16] Suganya S, Sankareshwari B (2020). Job Satisfaction Level on Online Teaching among Higher Secondary School Teachers during COVID-19 Pandemic. Shanlax International Journal of Education.

[17] Dabrowski A (2021). Teacher wellbeing during a pandemic: surviving or thriving?. Soc Educ Res.

[18] Federkeil L, Heinschke F, Jungmann T, Klapproth F (2020). Teachers Experiences of Stress and Their Coping Strategies During COVID-19 Induced Distance Teaching. J Pedagog Res.

[19] Lizana PA, Vega-Fernadez G, Gomez-Bruton A, Leyton B, Lera L (2021). Impact of the COVID-19 Pandemic on Teacher Quality of Life: A Longitudinal Study from Before and During the Health Crisis. Int J Environ Res Public Health.

[20] Ravens-Sieberer U, Kaman A, Erhart M, Devine J, Schlack R, Otto C (2021). Impact of the COVID-19 pandemic on quality of life and mental health in children and adolescents in Germany. Eur Child Adolesc Psychiatry.

[21] (2017). Supporting Learners with Healthcare Needs.

[22] Hopkins L, Green J, Henry J, Edwards B, Wong S (2014). Staying engaged: the role of teachers and schools in keeping young people with health conditions engaged in education. Aust Educ Res.

[23] Wilkie KJ. (2012). ‘Absence Makes the Heart Grow Fonder’: Students with Chronic Illness Seeking Academic Continuity Through Interaction With Their Teachers at School. Australasian Journal of Special Education.

[24] Filipušić I, Opić S, Krešić V (2015). Some Specificities of Students' Satisfaction with Hospital School. Croat J Educ.

[25] St Leger P (2014). Practice of Supporting Young People with Chronic Health Conditions in Hospital and Schools. Int J Incl Educ.

[26] Guleryuz OD, Hasirci D (2018). Children’s Hospital Schools as Social Environments: A Turkish Example. INTED2018 Proceedings;.

[27] Benigno V, Fante C (2020). Hospital School Teachers’ Sense of Stress and Gratification: An Investigation of the Italian Context. Contin Educ.

[28] Wilks SA (2010). A Charter for Children's Learning at the Royal Children's Hospital: Literature Review.

[29] Irwin MK, Elam M (2011). Are We Leaving Children with Chronic Illness Behind?. Phys Disabil Educ Relat Serv.

[30] Steinke SM, Elam M, Irwin MK, Sexton K, McGraw A (2016). Pediatric Hospital School Programming: An Examination of Educational Services for Students Who Are Hospitalized. Phys Disabil Educ Relat Serv.

[31] Cousin G (2009). Researching Learning in Higher Education: An Introduction to Contemporary Methods and Approaches.

[32] Blandford AE, Soegaard M, Dam R (2013). The Encyclopedia of Human-Computer Interaction. 2nd Edition.

[33] Äärelä T, Määttä K, Uusiautt S (2016). Ten Encounters between Students and a Special Education Teacher at a Finnish Hospital School–Outlining Hospital School Pedagogy. Global Journal of Human-Social Science.

[34] Mintz J, Palaiologou I, Carroll C (2018). A Review of Educational Provision for Children Unable to Attend School for Medical Reasons.

[35] Santamaría MD, Mondragon NI, Santxo NB, Ozamiz-Etxebarria N (2021). Teacher stress, anxiety and depression at the beginning of the academic year during the COVID-19 pandemic. Glob Ment Health.

[36] Collie RJ (2021). COVID-19 and Teachers’ Somatic Burden, Stress, and Emotional Exhaustion: Examining the Role of Principal Leadership and Workplace Buoyancy. AERA Open.

[37] Keehan S (2021). Continuing Education in Irish Hospital Schools: Provision for and Challenges for Teachers. Contin Educ.

[38] Benigno V, Dagnino F, Fante C (2020). Exploring the Impact of the COVID-19 Pandemic on Italy’s School-in-Hospital (SiHo) Services: The Teachers’ Perspective. Contin Educ.

[39] Äärelä T, Määttä K, Uusiautti S (2018). The Challenges of Parent–Teacher Collaboration in the Light of Hospital School Pedagogy. Early Child Dev Care.

[40] Ratnapalan S, Rayar MS, Crawley M (2009). Educational Services for Hospitalized Children. Paediatrics and Child Health.

[41] Małkowska-Szkutnik A, Berkowska A, Gajda M, Kleszczewska D (2021). Teaching in Hospitals and Healthcare Resorts: A Qualitative Study of Teachers’ Needs. Educ. Sci.

[42] Marshall DT, Shannon DM, Love SM (2020). How Teachers Experienced the COVID-19 Transition to Remote Instruction. Phi Delta Kappan.

